# Peripheral Blood Immune Cell Composition After Autologous MSC Infusion in Kidney Transplantation Recipients

**DOI:** 10.3389/ti.2023.11329

**Published:** 2023-06-23

**Authors:** Sanne H. Hendriks, Sebastiaan Heidt, Axel R. Schulz, Johan W. de Fijter, Marlies E. J. Reinders, Frits Koning, Cees van Kooten

**Affiliations:** ^1^ Department of Immunology, Leiden University Medical Center (LUMC), Leiden, Netherlands; ^2^ German Rheumatism Research Center (DRFZ), Berlin, Germany; ^3^ Department of Internal Medicine (Nephrology) and Transplant Center, Leiden University Medical Center, Leiden, Netherlands; ^4^ Department of Internal Medicine, Nephrology and Transplantation, Erasmus MC Transplant Institute, Erasmus University Medical Center, Leiden, Netherlands

**Keywords:** kidney transplantation, immunosuppression, mesenchymal stromal cells, immune regulation, mass cytometry

## Abstract

Tacrolimus is the backbone of immunosuppressive agents to prevent transplant rejection. Paradoxically, tacrolimus is nephrotoxic, causing irreversible tubulointerstitial damage. Therefore, infusion of mesenchymal stromal cells (MSC) 6 and 7 weeks post-transplantation was assessed to facilitate withdrawal of tacrolimus in the randomized phase II TRITON trial. Here, we performed detailed analysis of the peripheral blood immune composition using mass cytometry to assess potential effects of MSC therapy on the immune system. We developed two metal-conjugated antibody panels containing 40 antibodies each. PBMC samples from 21 MSC-treated patients and 13 controls, obtained pre-transplant and at 24 and 52 weeks post-transplantation, were analyzed. In the MSC group at 24 weeks, 17 CD4^+^ T cell clusters were increased of which 14 Th2-like clusters and three Th1/Th2-like clusters, as well as CD4+FoxP3+ Tregs. Additionally, five B cell clusters were increased, representing either class switched memory B cells or proliferating B cells. At 52 weeks, CCR7^+^CD38^+^ mature B cells were decreased. Finally, eight Tc1 (effector) memory cytotoxic T cell clusters were increased. Our work provides a comprehensive account of the peripheral blood immune cell composition in kidney transplant recipients after MSC therapy and tacrolimus withdrawal. These results may help improving therapeutic strategies using MSCs with the aim to reduce the use of calcineurin inhibitors.

**Clinical Trial Registration**: ClinicalTrials.gov, identifier NCT02057965.

## Graphical Abstract

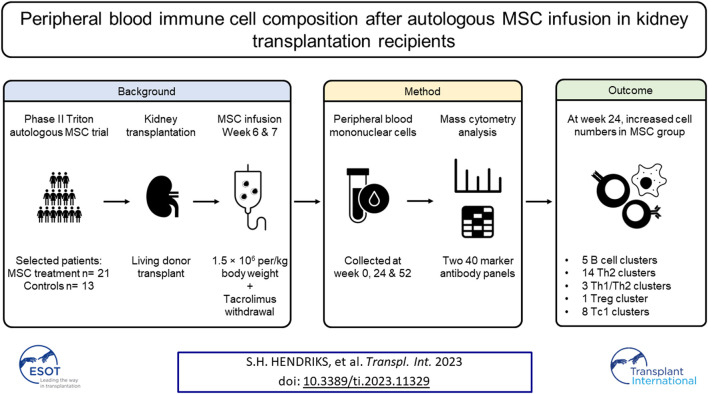

## Introduction

Kidney transplantation remains the preferred treatment for end-stage renal disease [1]. Tacrolimus, a calcineurin inhibitor, is the backbone of immunosuppressive protocols after kidney transplantation. Together with other immunosuppressive agents tacrolimus has vastly improved short-term allograft survival. However, despite these significant improvements, long-term kidney graft survival has not improved accordingly, partly due to long-term toxicity of immunosuppressive drugs [2–4]. Notably, tacrolimus is nephrotoxic, causing irreversible tubulointerstitial damage, which has led to numerous attempts to wean tacrolimus from the immunosuppressive regimen [5]. However, several studies have shown that tacrolimus withdrawal led to acute rejection episodes even in long-term stable patients [6–8]. Therefore, novel therapies are necessary to improve long term graft survival and minimize side effects of the current regiments.

One such new strategy that may allow cessation of tacrolimus use is mesenchymal stromal cell (MSC) therapy. MSCs have been shown to exert anti-inflammatory, immune-regulatory and tissue repair properties [9, 10]. They can interact both directly and indirectly with various immune cells [9, 10]. However, due to the observed short lifespan of MSCs *in vivo* [11], indirect effects through the release of extracellular vesicles, membrane particles and by undergoing apoptosis are thought to be most prominent. As such, MSC-derived vesicles may trigger monocytes and phagocytes to induce tolerogenic dendritic cells and regulatory T cells (Tregs) [11, 12]. This makes MSCs a promising new option to allow for tacrolimus weaning after kidney transplantation and possibly even the induction of immunological tolerance.

In the randomized phase II TRITON trial, administration of autologous bone marrow derived MSCs with concomitant early tacrolimus withdrawal was compared to standard tacrolimus dosing in living-donor kidney transplant recipients [13, 14]. The MSC group received MSC infusion at week 6 and 7, after which tacrolimus was reduced by half at week 7 and completely withdrawn at week 8. The control group remained on standard tacrolimus dosing and the study was performed using alemtuzumab as induction and an mTOR inhibitor as maintenance therapy. In our previous work, using flow cytometry on freshly obtained samples, we showed an increase in absolute number of peripheral blood Tregs in the MSC group compared to controls at 24 and 52 weeks after transplantation [13].

In this study we applied mass cytometry to perform in-depth characterization of the peripheral blood immune composition of patients included in the TRITON trial. We developed and validated two metal-conjugated mass cytometry antibody panels containing 40 antibodies each for the staining of bio-banked PBMCs and studied the influence of MSC therapy on immune cell subsets at 24 and 52 weeks after transplantation.

## Materials and Methods

### Study Design

The TRITON clinical trial was a randomized phase II, prospective, single-center, open-label study in living-donor kidney transplant recipients in which autologous bone marrow derived MSC therapy, with concomitant early tacrolimus withdrawal, was compared to standard tacrolimus dosing. The study was performed at the Leiden University Medical Center (LUMC), the Netherlands. The trial design and trial protocol have been previously described and were approved by the local ethics committee at the LUMC, Leiden, and by the Central Committee on Research involving Human Subjects in the Netherlands [13, 14]. The trial was performed in accordance with the principles of the Declaration of Helsinki. Inclusion and exclusion criteria were described in the trial protocol [14]. Written informed consent was obtained from all participants.

In short, patients in the MSC group received two doses of autologous bone marrow derived MSCs, intravenously at weeks 6 and 7 after transplantation. Bone marrow was aspirated from the posterior iliac crest of all patients in the MSC group during the renal transplantation. Processing of the MSCs took place at the GMP Facility of the LUMC. The MSC product was infused at week 6 and week 7 via peripheral infusion within 30 min with a target dose of 1.5 × 106 per/kg body weight intra venously (range 1–2 × 10^6^ cells).

During the trial, protocol blood samples were obtained before transplantation (week 0), at weeks 6, 12, 24 and 52 after transplantation. Of the 70 subjects, 34 were selected for the mass cytometry study of which 21 had received MSC treatment and 13 were control patients. Selection was based on the availability of sufficient PBMCs, a 3:2 ratio between the MSC group and control group and similar age distribution (control; 26–66 years, mean: 50 years, MSC; 31–70 years, mean: 51 years), Supplementary Table S1.

All patients received their allocated treatment. In the control group one patient had not enough PBMCs stored at 24 weeks and another patient lacked the 52 weeks timepoint. Limited immune phenotyping by flow cytometry was already performed on fresh PBMCs of these patients and showed several differences at 24 and 52 weeks [13]. Therefore, we selected week 0, week 24 and week 52 for high dimensional analysis by mass cytometry.

### Mass Cytometry Staining and Data Acquisition

PBMCs were isolated by Ficoll-Paque density-gradient centrifugation and cryopreserved in liquid nitrogen until time of analysis in RPMI, 20%FCS, 10%DMSO. Two metal conjugated 40-antibody panels for mass cytometry were developed, panel 1 focusing on B cell, NK cell and T cell markers and panel 2 focusing on myeloid and NK cell markers. Heavy metal isotope-tagged monoclonal antibodies (mAbs) for mass cytometry are listed in Supplementary Tables S2, S3. Samples were live-cell barcoded, stained and measured in batches of nine patient samples and one reference sample (total of 11 batches, samples of each patient were kept within one batch). Barcoding of live cell samples was performed with α-B2M (anti-β-2-microglobulin) and α-CD298 mAbs using a protocol adapted from Mei et al [15]. In brief, both mAbs were conjugated to Pd104, Pd105, Pd106, Pd108 or Pd110 using isothiocyanobenzyl-EDTA. Next, 10 barcode mixes were made, each containing both α-B2M and α-CD298 conjugated to their respective Pd isotopes, aliquoted and stored at −80°C until time of staining. For staining, purified mAbs were pre-conjugated by Fluidigm or conjugated with heavy metals in-house using the MaxPar X8 Antibody Labeling Kitaccording to the manufacturer’s instructions (Fluidigm). All mAbs were titrated to determine the optimal labelling concentration. Antibody mixes for barcoding and extracellular staining of panel 1 and panel 2 were aliquoted in maxpar cell staining buffer, the intracellular mix of panel 1 was aliquoted in Perm Buffer (eBiosciences), all stored at −80°C until time of staining.

PBMCs were thawed, washed with RPMI, 50%FCS, and incubated with 0.04 mg/mL DNase in IMDM, 10% FCS in at room temperature (RT) for 30 min. Cells were washed with IMDM, 10% FCS, counted and for each panel 2.5*10^6^ cells/sample were washed with cell staining buffer. Next, the cells were incubated with 1 mL cell staining buffer containing 1 μM Cell-ID intercalator-103Rh (Fluidigm) for 15 min at RT. Cells were washed and incubated with human Fc receptor block (BioLegend) for 10 min at RT and stained with thawed barcode antibody mixes for 45 min at RT. After washing twice, 10 samples were pooled, washed and incubated for 45 min at RT with the extracellular antibody mix. After washing, for panel 1 intracellular staining was performed, for panel 2 we continued with DNA staining. Intracellular staining was performed using the Foxp3/transcription factor staining buffer set (eBiosciences). Cells were incubated with Fix/Perm working solution for 45 min at 4°C, cells were washed with Perm Buffer and incubated with thawed intracellular antibody mix for 30 min at RT in a final volume of 200 µL. For the DNA stain, the cells were washed, incubated with 1 mL Maxpar Fix and Perm buffer (Fluidigm) containing 0.125 μM Cell-ID intercalator-Ir (Fluidigm) overnight at 4°C.

Cells were acquired within 48 h of staining on a Helios mass cytometer (Fluidigm) at an event rate of <250 events/sec in Cell Acquisition Solution containing ×10 diluted EQ Four Element Calibration Beads (Fluidigm). For the compensation matrix, staining beads (eComp) were individually stained with the conjugated mAbs and incubated for 45 min. After washing, the beads were pooled, washed and acquired in cell staining buffer. Experiments and acquisition were performed in a period of 81 days.

### Mass Cytometry Data Analysis

Data were normalized with EQ-normalization passport for each experiment. Subsequently, the data were gated using Flowjo v10.6.1, using channels 89Y_CD45, 193Ir_DNA, Residual, 103Rh_DNA (live/dead) and 140Ce_bead, removing debris, dead cells and doublets. Next, the data were compensated and debarcoded in R v4.1.1 using the CATALYST package and automatic cutoffs. The data were arcsin 5 transformed in Cytosplore. Using the reference sample, the data were corrected for batch effects using R after which the data were downsampled to a maximum of 50.000 cells/sample. For the discovery analysis the FlowSOM package was used [16]. The downsampled cells were used for the first overview FlowSOM, containing 100 clusters gathered in 30 metaclusters. Next, metaclusters sharing similar phenotypes were merged, resulting in four groups (panel 1) and three groups (panel 2). A separate FlowSOM was performed for each group, allowing for in-depth analysis. For panel 1, group 1, 2 and 3, and panel 2, group 1, 2 and 3, a FlowSOM was created with 121 clusters and 100 metaclusters and for panel 1 group 4, a FlowSOM with 225 clusters and 200 metaclusters was made. Metaclusters with similar phenotypes were merged, resulting clusters contained all >500 cells and originated from different samples. Doublet clusters were removed. Using absolute cell counts obtained on fresh blood samples using the BD Multitest kit (BD Biosciences) the absolute number of cells per cluster were calculated. Finally, for each cluster, the MSC therapy group was compared to the control group and graphs were made using Graphpad prism v8.4.2. Measurements with value 0 are depicted as a dot on the X-axis. For validation purposes selected subsets were gated using Flowjo v10.6.1.

### Statistical Analysis

For the discovery analysis, the comparisons of the control group versus the MSC therapy group within each cluster were performed with the Mann-Whitney U test in Graphpad prism version 8.4.2, corrected with Bonferroni.

## Results

### CD4^+^ T Cells are Increased in MSC-Treated Patients at 24 Weeks

**FIGURE 1:**
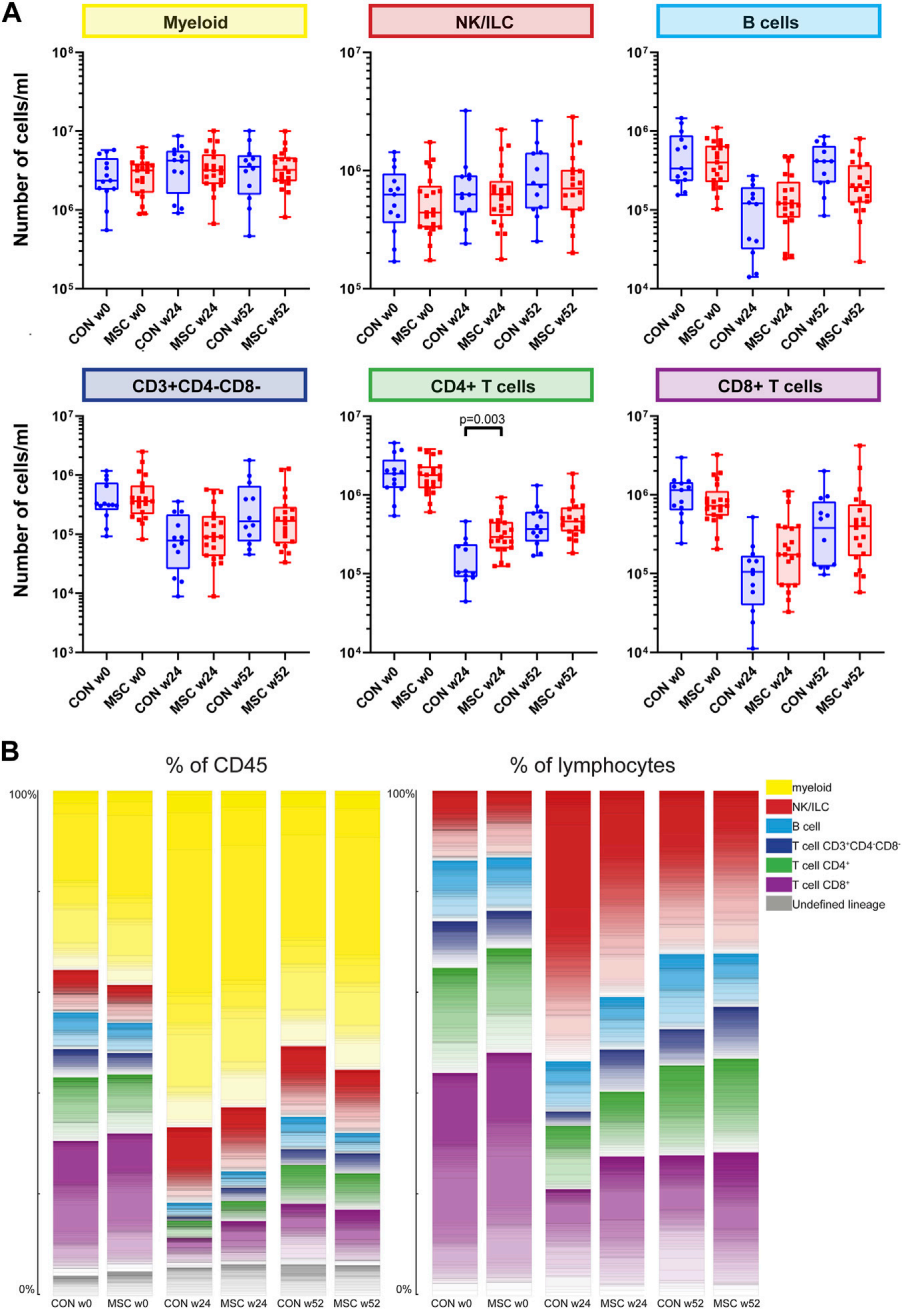
Major immune lineages. **(A)** Graphs showing the number of cells/mL in the control group and the MSC therapy group at timepoint w0, w24, and w52, for the five major immune lineages, Myeloid, NK/ILC, B cells, CD4^+^ T cells and CD8^+^ T cells. Each dot represents an individual patient within that timepoint. CON, control group (Blue); MSC, MSC therapy group (Red). P-values were calculated with the Mann-Whitney U test and corrected within each cluster with Bonferroni. **(B)** The contribution of the different cell clusters and major lineages as percentage of CD45^+^ cells (left panel) and as percentage of lymphocytes (right panel). Both for the control group and the MSC therapy group at timepoint w0, w24, and w52.

Data were analyzed using the FlowSOM clustering method. First, we performed a highly detailed analysis of panel 1 at the single cell level which resulted in the identification of 346 phenotypically distinct cell clusters. For each cluster we determined the major lineage (B cells, myeloid cells, CD4^+^ T cells, CD8^+^ T cells and NK/ILCs, Supplementary Figure S1) based on all markers in the panel, and compared the number of cells of each lineage in the control and the MSC therapy group at weeks 0, 24 and 52 (Figure 1A).

As a consequence of the alemtuzumab-induced lymphodepletion, B cells and T cells were still repopulating at week 24 and did not reach baseline levels at week 52, reflected in the data by a rising number of B cells and T cells between week 24 and week 52 in both groups. There was no difference in absolute cell numbers between the control and the MSC group for B cells, myeloid cells, NK/ILCs, CD3^+^CD4^−^CD8^−^ T cells and CD8^+^ T cells. However, at week 24 CD4^+^ T cells were increased in the MSC group compared to the control group (*p* = 0.003). To validate this finding, traditional two-dimensional manual gating was used for analysis of the major immune cell lineages. This likewise identified a significant increase of the CD4^+^ T cells in the MSC patients at week 24 (*p* = 0.038, Supplementary Figure S2).

Next, the contribution of each lineage as a percentage of the total CD45 population at each of the timepoints was assessed (Figure 1B). While pre-transplantation (week 0) lymphocytes make up 56%–60% and myeloid cells 35%–38% of the total CD45^+^ population, at week 24 this distribution was skewed towards a dominance of myeloid cells due to the alemtuzumab-induced lymphodepletion. As expected, at week 52 the myeloid cells still made up the majority of CD45^+^ cells, however the proportion of lymphoid cells was increasing. At 24 weeks, both the percentage of CD3^+^CD4^−^CD8^−^ and CD8^+^ cells were increased in the MSC group compared to the controls. While absolute cell numbers CD4^+^ T cells were increased in the MSC group compared to the controls, as a percentage of both total CD45^+^ cells and total lymphoid cells the numbers of CD4^+^ cells were similar in both groups (Figure 1B).

Investigating the 346 phenotypically distinct cell clusters individually, 33 (of which 32 assigneable to a lineage) showed a statistically significant difference in absolute cell numbers between the control and the MSC-treated group. The 32 lineage defined clusters will be further discussed below (Supplementary Table S4).

### Changes Within the B Cell Compartment Upon MSC Treatment

**FIGURE 2:**
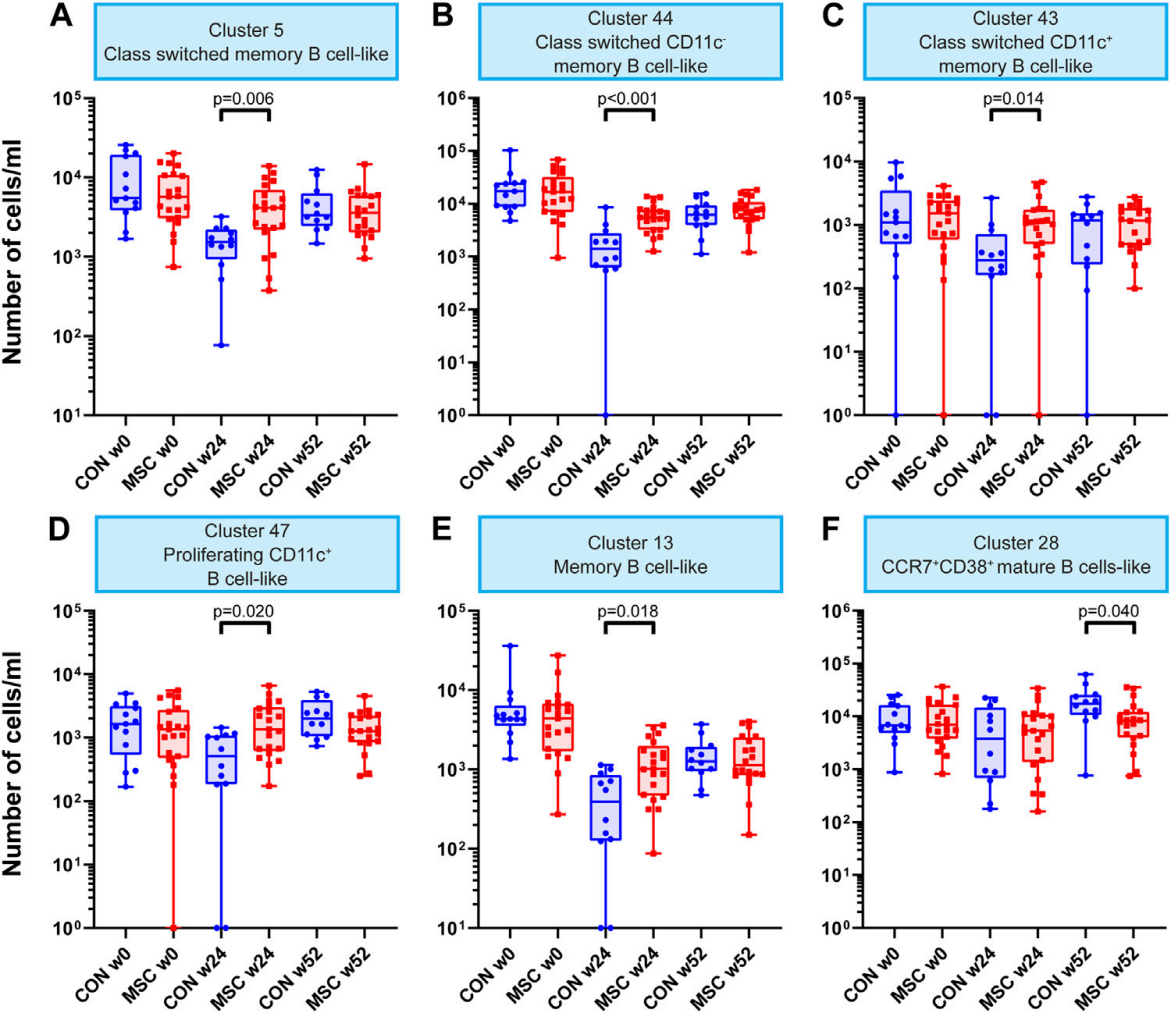
B cell clusters. Graphs showing the number of cells/mL at timepoint w0, w24, and w52 for both the control and the MSC therapy group. **(A)** Cluster 5; Class switched memory B cell-like. **(B)** Cluster 44; Class switched CD11c memory B cell-like. **(C)** Cluster 43; Class switched CD11c^+^ memory B cell-like. **(D)** Cluster 47; Proliferating CD11c^+^ B cell-like. **(E)** Cluster 13; Memory B cell-like. **(F)** Cluster 28; CCR7^+^CD38^+^ mature B cells-like. CON, control group (Blue); MSC, MSC therapy group (Red). P-values were calculated with the Mann-Whitney U test and corrected within each cluster with Bonferroni.

Within the B cell clusters (*n* = 47), five were increased in absolute cell numbers in the MSC therapy group compared to the control group at week 24 (Supplementary Table S4). These B cell clusters included class switched memory B cells, class switched CD11c^−^ and CD11c^+^ memory B cell-like clusters, a proliferating (Ki-67^+^) CD11c^+^ B cell-like cluster and a memory B cell cluster (Figures 2A–E). While these clusters were increased in MSC-treated patients at 24 weeks, they were similar to controls at 52 weeks. The sixth B cell cluster of CCR7^+^CD38^+^ mature B cells showed a decrease in absolute number of cells at week 52 in the MSC group compared to the control group (Figure 2F).

### Tc1-Like and Tc1/Tc2-Like Clusters are Enriched in MSC-Treated Patients at Week 24

**FIGURE 3:**
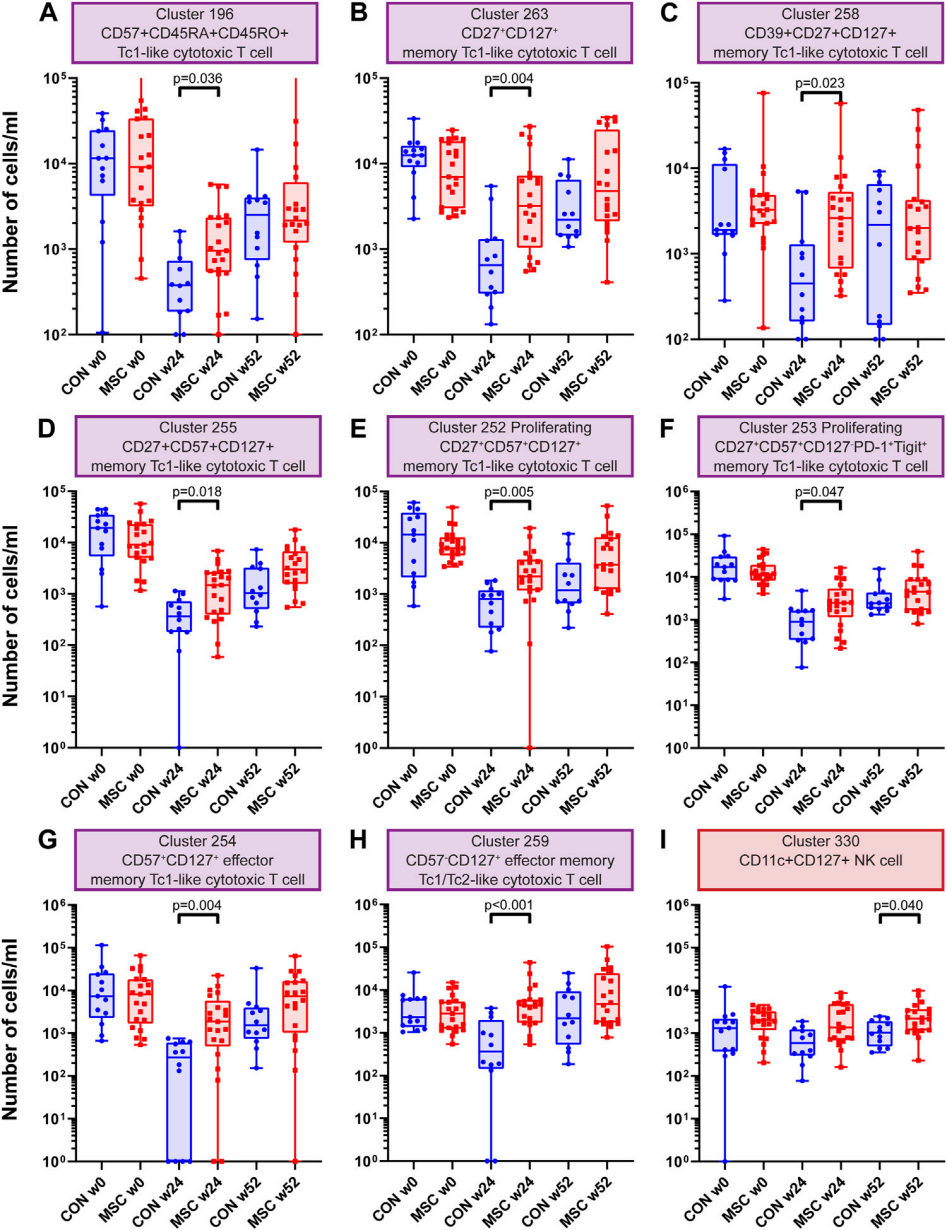
CD8 T cell and NK cell clusters. Graphs showing the number of cells/ml at timepoint w0, w24 and w52 for both the control and the MSC therapy group. **(A)** Cluster 196; CD57^+^CD45RA^+^CD45RO^+^ Tc1-like cytotoxic T cell. **(B)** Cluster 263; CD27^+^CD127^+^ memory Tc1-like cytotoxic T cell. **(C)** Cluster 258; CD39^+^CD27^+^CD127^+^ memory Tc1-like cytotoxic T cell. **(D)** Cluster 255; CD27^+^CD57^+^CD127^+^ memory Tc1-like cytotoxic T cell. **(E)** Cluster 252; Proliferating CD27^+^CD57^+^CD127^+^ memory Tc1-like cytotoxic T cell. **(F)** Cluster 253; Proliferating CD27^+^CD57^+^CD127^−^PD-1^+^Tigit^+^ memory Tc1-like cytotoxic T cell. **(G)** Cluster 254; CD57^+^CD127^+^ effector memory Tc1-like cytotoxic T cell. **(H)** Cluster 259; CD57^−^CD127^+^ effector memory Tc1/Tc2-like cytotoxic T cell. **(I)** Cluster 330; CD11c^+^CD127^+^ NK cell. CON: control group (Blue), MSC: MSC therapy group (Red). *p*-values were calculated with the Mann-Whitney U test and corrected within each cluster with Bonferroni.

Investigating the CD8^+^ T cells clusters (*n* = 73), eight clusters showed a statistically significant increase at week 24 in the MSC group compared to the control group (Supplementary Table S4). These were a CD57^+^CD45RA^+^CD45RO^+^ Tc1-like cytotoxic T cell cluster, three memory Tc1-like cytotoxic T cell clusters (CD27^+^CD127^+^, CD39^+^CD27^+^CD127^+^ and CD27^+^CD57^+^CD127^+^), two proliferating memory Tc1-like cytotoxic T cell clusters (CD27^+^CD57^+^CD127^+^ and CD27^+^CD57^+^CD127^−^PD-1^+^Tigit^+^), an CD57^+^CD127^+^ effector memory Tc1-like cytotoxic T cell cluster and finally an CD57^−^CD127^+^ effector memory Tc1/Tc2 cytotoxic T cell cluster (Figures 3A–H).

### Increased Numbers of CD11c^+^CD127^+^ NK Cells at 52 Weeks in the MSC Treated Patients

One NK cell cluster was increased in absolute cell numbers in the MSC therapy group compared to the control group at 52 weeks. This cluster is a CD11c^+^CD127^+^ NK cell cluster (Supplementary Table S4 and Figure 3I). NK cells are dominant in the early repopulation phase after alemtuzumab. Whereas the first antibody panel was able to discriminate between the major lineages, it did not contain highly detailed information about the different subsets of myeloid and NK cells. To get a more detailed insight into the NK cells and myeloid cells, we developed a second antibody panel. The discovery FlowSOM analysis of this panel resulted in a total of 85 phenotypically distinct myeloid and NK/ILC clusters of which only cluster 15 was significantly different between the control group and MSC treated patients. However, this is unlikely to be due to the treatment, since this difference was already present pre-transplantation (Supplementary Figure S3).

### Cell Numbers of Th2-Like and Th1/Th2-Like Clusters are Elevated in MSC-Treated Patients at Week 24

Exploring the CD4^+^ T cell clusters (*n* = 57), 17 showed a statistically significant increase in absolute cell numbers at week 24 in the MSC group compared to the control group (Table 1, Supplementary Table S4). These were a CD7^+^CD27^+^CD127^+^ central memory Th2-like cluster, seven effector memory Th2-like clusters, six activated effector memory Th2-like clusers and three effector memory Th1/Th2-like clusters.

**TABLE 1:** CD4 clusters.

Cluster	Cell type	Cells/μL median (range) week 0			Cells/μL median (range) week 24			Cells/μL median (range) week 52		
		Control	MSC	*p*-value	Control	MSC	*p*-value	Control	MSC	*p*-value
	**CD4 central memory Th2**									
141	CD7^+^CD27^+^CD127^+^ central memory Th2-like	412 (122–1,091)	371 (147–881)	ns	36 (13–158)	84 (27–230)	0.047	109 (33–291)	92 (34–236)	ns
	**CD4 effector memory Th2**									
149	CD7^+^CD27^+^CD127^+^ effector memory Th2-like	9 (1–34)	7 (3–51)	ns	1 (0–6)	3 (1–26)	0.005	1 (3–33)	1 (6–73)	ns
151	CD7^lo^CD27^+^CD127^+^ effector memory Th2-like	44 (20–122)	53 (21–151)	ns	2 (1–7)	5 (1–17)	0.038	7 (3–21)	9 (2–16)	ns
228	CD7^−^CD127^+^ effector memory Th2-like	15 (5–126)	16 (6–113)	ns	1 (0–6)	2 (0–14)	0.023	3 (1–44)	7 (1–39)	ns
224	CD127^+^CD161^+^ effector memory Th2-like	11 (3–43)	12 (2–27)	ns	1 (0–4)	2 (0–15)	0.030	3 (1–23)	5 (2–72)	ns
220	CD39^+^CD7^−^CD127^+^ effector memory Th2-like	4 (1–17)	3 (1–55)	ns	0 (0–8)	3 (1–49)	0.009	2 (1–61)	4 (0–87)	ns
152	CD27^+^CD127^−^PD-1^+^ effector memory Th2-like	5 (1–11)	4 (1–20)	ns	2 (1–7)	4 (1–25)	0.023	3 (1–19)	4 (1–27)	ns
96	Proliferating HLA-DR^+^CD7^−^CD127^+^ effector memory Th2-like	5 (1–20)	5 (1–40)	ns	1 (0–14)	3 (0–20)	0.011	4 (0–20)	4 (0–33)	ns
	**Activated CD4 effector memory Th2**									
229	CD7^+^CD127^+^ activated effector memory Th2-like	15 (3–73)	13 (4–106)	ns	1 (0–7)	3 (0–19)	0.008	4 (0–19)	6 (1–33)	ns
145	CD7^+^CD27^+^CD127^+^ activated effector memory Th2-like	10 (4–49)	13 (4–84)	ns	0 (0–5)	2 (0–17)	0.020	3 (0–18)	4 (1–29)	ns
148	CD7^−^CD27^+^CD127^+^ activated effector memory Th2-like	11 (3–55)	13 (6–64)	ns	1 (0–3)	3 (0–12)	<0.001	3 (1–14)	6 (1–21)	ns
226	CD7^−^CD127^+^ activated effector memory Th2-like	9 (3–23)	10 (4–37)	ns	1 (0–3)	3 (1–46)	0.014	5 (0–12)	4 (2–36)	ns
223	CD127^+^CD161^+^PD-1^+^ activated effector memory Th2-like	11 (1–27)	11 (2–57)	ns	2 (0–7)	4 (1–27)	0.005	6 (1–18)	5 (2–72)	ns
227	CD7^+^CD27^+^ activated effector memory Th2-like	28 (11–113)	38 (9–138)	ns	1 (0–6)	5 (0–22)	0.008	6 (1–32)	9 (2–50)	ns
	**CD4 effector memory Th1/Th2**									
217	CD57^+^CD127^+^PD-1^+^ effector memory Th1/Th2-like	2 (0–6)	2 (0–16)	ns	0 (0–4)	3 (0–65)	<0.001	2 (0–56)	7 (1–80)	ns
222	Proliferating CD57^+^CD127^+^ effector memory Th1/Th2-like	7 (1–37)	6 (2–27)	ns	1 (0–4)	3 (0–40)	0.030	4 (1–18)	8 (0–46)	ns
221	Proliferating HLA-DR^+^CD39^+^CD57^+^CD127^+^ effector memory Th1/Th2-like	3 (1–9)	2 (0–15)	ns	0 (0–8)	3 (0–13)	0.023	3 (1–41)	4 (0–70)	ns

Table depicting significant CD4 clusters. Control, control group; MSC, MSC therapy group; ns, not significant. P-values were calculated with the Mann-Whitney U test and corrected within each cluster with Bonferroni.

### Mass Cytometry Analysis Confirms Transient Increase in Treg Numbers in MSC-Treated Patients

**FIGURE 4:**
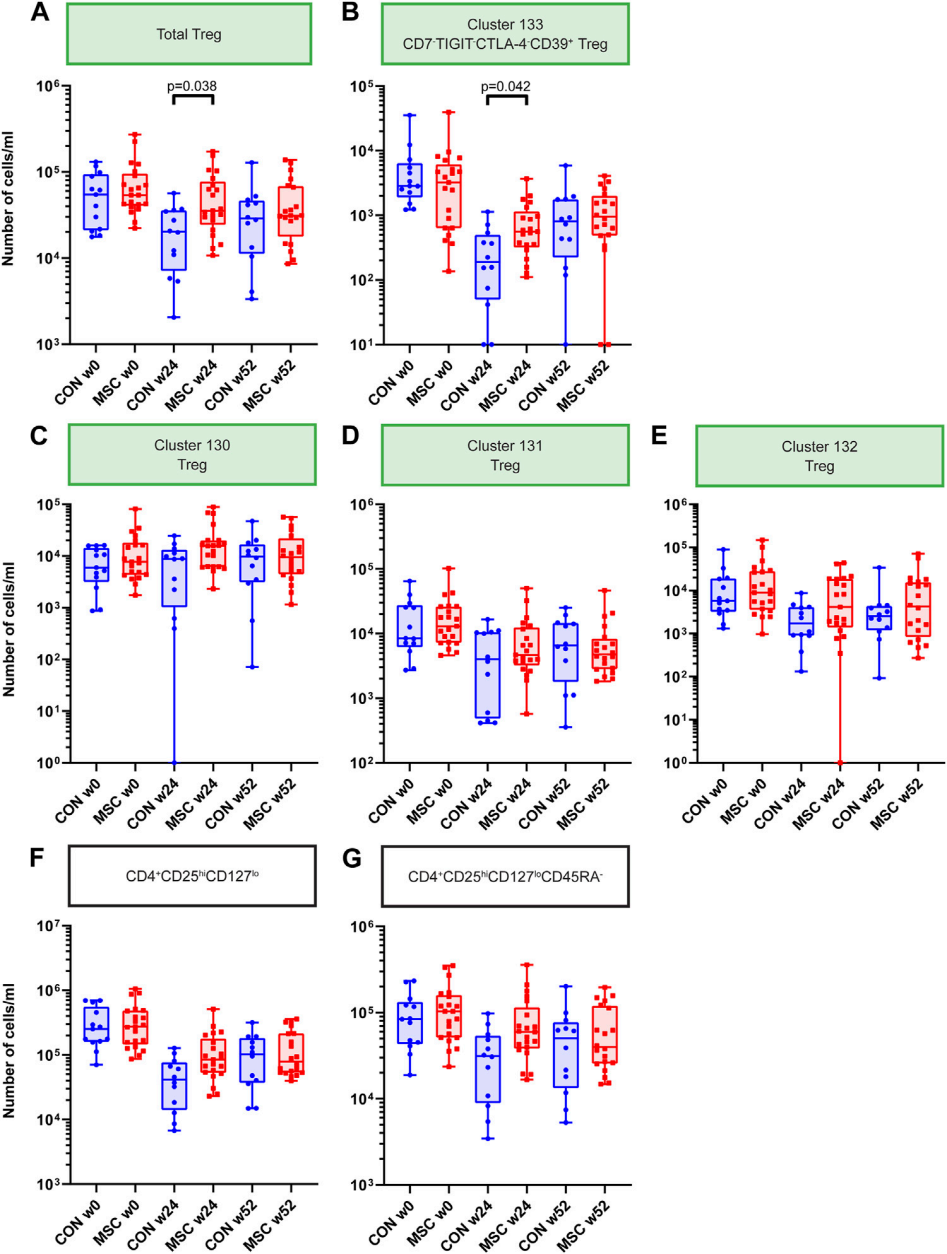
Treg Clusters. Graphs showing the number of cells/mL at timepoint w0, w24, and w52 for both the control and the MSC therapy group. **(A)** Total Treg. **(B)** Cluster 133; CD7-TIGIT-CTLA-4-CD39^+^ Treg. **(C)** Cluster 130; Treg. **(D)** Cluster 131; Treg. **(E)** Cluster 132; Treg. **(F)** CD4^+^CD25hiCD127lo subset after manual gating. **(G)** CD4^+^CD25hiCD127loCD45RA-subset after manual gating. CON, control group (Blue); MSC, MSC therapy group (Red). P-values were calculated with the Mann-Whitney U test and corrected within each cluster with Bonferroni.

In our analysis we could identify four FoxP3^+^ Treg clusters (clusters 130, 131, 132, and 133) which combined likely reflect the total pool of Tregs in the samples. We observed that the total Tregs were increased at 24 weeks for the MSC group (*p* = 0.038, Figure 4A). Also when evaluating the four Treg clusters individually, a similar increase in cell numbers at week 24 was observed for the MSC treated patients, reaching significance for cluster 133 (Figures 4B–E). Cluster 133, FoxP3^+^CD7^−^TIGIT^-^CTLA-4^−^CD39^+^, differed from the other 3 FoxP3+ Treg clusters by lack of CD7 and Tigit.

Previously, as part of the original TRITON study protocol, fresh PBMCs were measured with the ONE-Study flow cytometry panel [13, 17]. In these analyses absolute cell numbers of CD4^+^CD25^hi^CD127^lo^ and CD4^+^CD25^hi^CD127^lo^CD45RA^-^ Tregs were increased in MSC treated patients at week 24 and week 52. Therefore, in the current mass cytometry study, we used manual gating to identify and quantify these CD4^+^CD25^hi^CD127^lo^ and CD4^+^CD25^hi^CD127^lo^CD45RA^-^ subsets. This revealed a similar trend at week 24, although not reaching statistical significance (Figures 4F, G).

## Discussion

In this study we used mass cytometry to investigate the effect of the application of MSC therapy at week 6 and 7 after kidney transplantation with concomitant tacrolimus withdrawal on a background of alemtuzumab and mTOR inhibitor. In previous work we described successful tacrolimus withdrawal after MSC infusion [13]. Furthermore, we observed an increase of peripheral blood Tregs in the MSC group compared to controls at 24 and 52 weeks after transplantation [13]. In the current study we aimed to better understand the influence of MSCs on the immune system and on facilitating tacrolimus withdrawal. Two mass cytometry panels were developed, together covering 69 immune cell markers and used to determine the composition of the peripheral blood immune cell compartment of control patients and patients receiving MSC therapy.

MSCs can affect many types of immune cells including dendritic cells, monocytes, macrophages, B cells, T cells (Treg/Th1/Th2 and Th17 helper cells), NK cells and NKT cells, ILCs, myeloid-derived suppressor cells, neutrophils, and mast cells through a combination of direct cell-cell contact and soluble factors (reviewed by Weiss et al. and Jiang et al. [18, 19]). MSCs are incapable of passing narrow capillary networks due to their size and thus often accumulate in the lungs upon intravenous infusion [11, 20]. The additional short-life span makes direct cell-cell interaction in other tissues, such as the kidneys, unlikely in the context of a therapeutic setting. Immune modulatory and organ regenerative effects of MSCs can also be mediated by their secretome, as shown in various immune and injury models [21, 22]. Part of the secretome of MSCs are extracellular vesicles. These vesicles contain extracellular matrix proteins, cell adhesion proteins and microRNAs, all able to influence the immunological response [21, 23, 24]. To which degree these factors are important after therapeutic MSC infusion is still unclear. Finally, it has been shown that MSC therapy could affect the immune system as a result of apoptosis and subsequent phagocytosis by monocytes/macrophages, neutrophils and dendritic cells [10]. Upon phagocytosis these cells were shown to migrate through the bloodstream to the liver and other organs, with altered phenotype and function, possibly modulating the immune response over an extended period of time [11, 25]. In this study, we therefore extensively investigated changes in the myeloid compartment after MSC infusion. Our results showed no differences in this compartment, we could therefore not confirm (long term) involvement of myeloid cells.

In the current study alemtuzumab was used as induction therapy, resulting in profound depletion of circulating lymphocytes. As expected, we could confirm that repopulation after induction therapy is still ongoing at 52 weeks, as the number of CD4^+^ T cells and CD8^+^ T cells was still lower compared to baseline. At 24 weeks, we observed that the absolute numbers of CD4^+^ T cells were significantly higher in the MSC group compared to the control group, a difference which disappeared at week 52. This is in line with our previous work where we reported a increase of CD4^+^ T cells in the MSC group at 12 weeks and an a similar trend at 24 weeks [13]. This flow cytometry based approach did not allow for detailed analysis on the underlying T cell subsets, which the current mass cytometry based analysis did. Exploring the immune cell clusters in more detail, we found 30 clusters to be increased exclusively at week 24 in MSC treated patients (five B cell, 17 CD4^+^ T cell and eight CD8^+^ T cell clusters). In contrast, only three clusters, one NK cell, one lineage undefined and one B cell cluster were different between the treatment groups at 52 weeks. Among the 17 increased CD4^+^ T cell clusters in MSC treated patients were 14 Th2-like clusters (six activated) and three Th1/Th2-like clusters. Th2 cells can be primed by phagocytotic cells, like monocytes and dendritic cells, after engulfing MSCs. By releasing IL-4 and IL-10, Th2 cells can repress the development of the Th1 cells and subsequently repress an inflammatory environment [26]. We also observed increased cell numbers in eight Tc1 (effector) memory cytotoxic T cell clusters, which are considered to be highly inflammatory and if directed towards the transplanted kidney might play a role in transplant rejection. We also showed five B cell clusters increased in the MSC group at week 24. These clusters are either class switched memory B cells or proliferating B cells, indicating a possible increase in the capacity to produce antibodies in the MSC group. In line with this, while in the MSC group 7/21 patients developed dnDSA at week 24 none of the control patients (0/13) developed dnDSA. However, when splitting the MSC group into DSA+ and DSA− this did not result in significant differences in any of the 33 clusters discussed. When comparing the two groups at 52 weeks, all the above clusters contained similar cell numbers while cluster 28 (CCR7^+^CD38^+^ mature B cells), was decreased in the MSC group. Finally, Tregs have been proposed to be the mediators of the immune dampening effects of MSCs and were elevated in MSC treated patients when analyzed with flow cytometry on fresh PBMC samples [13]. In line with this, we could confirm the increase of Treg cells at week 24 in the MSC group in this study, indicating a immune dampening environment.

In the TRITON trial, MSCs were administered to facilitate safe tacrolimus withdrawal. Although the MSC patients with tacrolimus withdrawal developed more dnDSA then the control group, their kidney function was not inferior and dnDSA development did not lead to more rejection episodes [13, 27]. In our selection of patients from the TRITON study there was one patient in the control group with a rejection episode (mixed rejection) and two patients in the MSC group with a rejection episode (TCMR) within the first year after transplantation (Supplementary Table S1). This number of rejections is too low to correlate them with the discovered subsets. In the Triton study tacrolimus withdrawal was save. We observed that the MSC-treated patients had increased number of cells in multiple immune dampening Th2 subsets as well as an in-creased amount of Tregs. These subsets might play a role in the ability to safely withdraw tacrolimus in combination with MSC infusion. On the other hand, it is important to acknowledge that changes in immune cell subsets might be caused both by the MSC administration as well as by the tacrolimus withdrawal, an effect we cannot dissociate in the current study.

Tacrolimus inhibits the calcineurin pathway preventing dephosphorylation of NFAT (nuclear factor of activated T lymphocytes) and its translocation to the nucleus. This blocks the activation of the IL-2 gene, involved in T cell activation and as a result the initiation of the immune response [28]. In this light, the increased numbers of Th2 and Tc1 cells in MSC treated patients could be the result of the absence of tacrolimus. We therefore cannot determine whether the increased subsets are the result of the infused MSCs or of tacrolimus withdrawal. Also, as the specificity of the B cells and T cells is unknown, it is unclear if the increased subsets are directed against the transplanted kidney or if they are part of ongoing repopulation. Single-cell RNA sequencing including T cell and B cell receptor analysis could shed light on the clonality and specificity of these repopulating cells.

The analysis performed in this study was an unbiased discovery analysis using a large number of immune cell markers. Therefore, many phenotypically different clusters could be identified, and as a result a high number of comparisons were made. In this study we corrected with Bonferroni for the three comparisons made within one cluster. Correcting for false positives within the whole study, with the current statistical options, would result in the need for extremely low *p*-values to remain significant after correction. For this reason we propose that the subsets found in this study should be further investigated for their contributions to MSC therapy in a more focused analysis in future studies.

In conclusion, this study provides a comprehensive description of the PBMC subsets in kidney transplantation patients after MSC therapy and subsequent tacrolimus withdrawal. Our results point towards an active involvement of CD4^+^ Th2 cells, Tregs, class switched memory B cells and CD8^+^ Tc1 cells in patients receiving MSCs and the save practice of tacrolimus withdrawal. Future studies are required to validate these findings and investigate the possible functional role of the identified immune cell subsets.

## Data Availability

The raw data supporting the conclusion of this article will be made available by the authors, without undue reservation.

## Abbreviations

B2M, β-2-microglobulin; dnDSA, *de novo* donor-specific antibodies; ILC, innate lymphoid cell; mAbs, monoclonal antibodies; MSC, mesenchymal stromal cells; NFAT, nuclear factor of activated T lymphocytes; NK cell, Natural killer cell; PBMC, Peripheral blood mononuclear cell; RT, room temperature; Tc, cytotoxic T cell; Th, helper T cell; Treg, regulatory T cell.
