# Supplementary material for: Peripheral Blood Immune Cell Composition After Autologous MSC Infusion in Kidney Transplantation Recipients
Source: Transpl Int. 2023 Jun 23;36:11329. doi: 10.3389/ti.2023.11329 (PMC10326287; doi:10.3389/ti.2023.11329)
Supplement: Supplementary file 3 [file Table1.DOCX]

**Table S1. Patient characteristics**

|  | **Control** | **MSC** |
| --- | --- | --- |
| **Number of patients** | 13 | 21 |
|  |  |  |
| **Sex:** |  |  |
| F/M | 2/11 | 1/20 |
|  |  |  |
| **Age:** |  |  |
| Mean (range) | 50 (24-66) | 51 (31-70) |
|  |  |  |
| **dnDSA:** |  |  |
| At 24 weeks | 0 | 7 |
| At 52 weeks | 0 | 7 |
|  |  |  |
| **Rejection:** |  |  |
| At 24 weeks |  |  |
| ABMR | 0 | 0 |
| TCMR | 0 | 1 |
| Mixed rejection | 0 | 0 |
| No rejection | 13 | 20 |
|  |  |  |
| At 52 weeks |  |  |
| ABMR | 0 | 0 |
| TCMR | 0 | 2* |
| Mixed rejection | 1 | 0 |
| No rejection | 12 | 19 |

*Same patient as at 24 weeks.
