# Supplementary material for: Peripheral Blood Immune Cell Composition After Autologous MSC Infusion in Kidney Transplantation Recipients
Source: Transpl Int. 2023 Jun 23;36:11329. doi: 10.3389/ti.2023.11329 (PMC10326287; doi:10.3389/ti.2023.11329)
Supplement: Supplementary file 4 [file Table2.DOCX]

**Table S2. Antibody panel 1**

|  | Antigen | Tag | Clone | Com. | Cat# | Lot# | Dilution |
| --- | --- | --- | --- | --- | --- | --- | --- |
| 1 | CD45 | 89y | HI30 | FLM | 3089003B | 3089003B | 1:100 |
| 2 | HLA-DR | 115ln | L243 | BioL | 307651 | B293412 | 1:100 |
| 3 | CCR6 (CD196) | 141 Pr | G034E3 | FLM | 3141003A | 0902010 | 1:100 |
| 4 | CD19 | 142 Nd | HIB19 | FLM | 3142001B | 0141921 | 1:200 |
| 5 | CD5 | 143 Nd | UCHT2 | FLM | 3143007B | 0451808 | 1:200 |
| 6 | CD69 | 144 Nd | FN50 | FLM | 3144018B | 1571908 | 1:200 |
| 7 | CD4 | 145 Nd | RPA-T4 | FLM | 3145001B | 2681902 | 1:100 |
| 8 | CD8 | 146 Nd | RPAT8 | FLM | 3146001B | 0861909 | 1:100 |
| 9 | CD40 | 147 Sm | 5C3 | BioLegend | 334325 | B254704 | 1:200 |
| 10 | PD-L1 | 148 Nd | 29E.2A3 | FLM | 3148017B | 0651910 | 1:200 |
| 11 | CD25  (IL2-R) | 149 Sm | 2A3 | FLM | 3149010B | 0161910 | 1:100 |
| 12 | CD57 | 150 Nd | HNK-1 | BioLegend | 359602 | B256178 | 1:200 |
| 13 | TCRgd | 152 Sm | 11F2 | FLM | 3152008B | 1622012 | 1:50 |
| 14 | CD7 | 153 Eu | CD7-6B7 | FLM | 3153014B | 0282010 | 1:100 |
| 15 | Tigit | 154 Sm |  | FLM | 3154016B | 0452004 | 1:100 |
| 16 | CD27 | 155 Gd | L128 | FLM | 3155001B | 1731805 | 1:200 |
| 17 | CXCR3 | 156 Gd | G025H7 | FLM | 3156004B | 2691901 | 1:100 |
| 18 | CD22 | 157 Gd | HIB22 | BioLegend | 302511 | B214802 | 1:400 |
| 19 | CD3 | 158 Gd | UCHT1 | BioLegend | 300443 | B289272 | 1:400 |
| 20 | CCR7 (CD197) | 159 Tb | G043H7 | FLM | 3159003A | 0912008 & 1402001 | 1:100 |
| 21 | KLRG-1 | 161 Dy | REA261 | MACS | 120-014-229 | 5190307174 | 1:100 |
| 22 | CD11c | 162 Dy | Bu15 | FLM | 3162005B | 0041909 | 1:200 |
| 23 | CD39 | 163 Dy | A1 | BioLegend | 328221 | B242203 | 1:200 |
| 24 | CD161 | 164 Dy | HP-3G10 | FLM | 3164009B | 0501901 | 1:100 |
| 25 | CD127 (IL-7Ra) | 165 Ho | A019D5 | FLM | 3165008B | 2541802 | 1:100 |
| 26 | IgM | 166 Er | MHM-88 | BioLegend | 314527 | B208787 | 1:400 |
| 27 | CD38 | 172 Yb | HIT2 | FLM | 3172007B | 0861906 | 1:200 |
| 28 | CD45RO | 173 Yb | UCHL1 | BioL | 304239 | B256595 | 1:100 |
| 29 | CD21 | 174 Yb | B-ly-4 | BD | 555421 | 9056779 | 1:200 |
| 30 | PD-1 | 175 Lu | EH 12.2H7 | FLM | 3175008B | 3451808 | 1:100 |
| 31 | CD56 | 176 Yb | NCAM16.2 | FLM | 3176008B | 0212012 | 1:100 |
| 32 | CD45RA | 198 Pt | HI100 | BioLegend | 304143 | B186475 | 1:200 |
| 33 | CD11b | 209Bi | ICRF44 | FLM | 3209003B | 1322021 | 1:100 |
| 34 | FoxP3 | 151 Eu | 259D/C7 | BD | 560044 | 9322626 | 1:100 |
| 35 | Tbet | 160 Gd | 4B10 | FLM | 3160010B | 3151304 & 3181905 | 1:50 |
| 36 | GATA-3 | 167 Er | TWAJ | FLM | 3167007A0 | 0592011 & 0391808 | 1:100 |
| 37 | Ki-67 | 168 Er | B56 | FLM | 3168007B | 0581816 | 1:50 |
| 38 | EOMES | 169 Tm | WD1928 | eBioscience | 14-4877-82 | 1944573 | 1:300 |
| 39 | CTLA-4 (CD152) | 170 Er | 14D3 | FLM | 317005B | 2621904 | 1:100 |
| 40 | CD40L (CD154) | 171 Yb | 24-31 | BioLegend | 310835 | B232943 | 1:200 |
