# Supplementary material for: Peripheral Blood Immune Cell Composition After Autologous MSC Infusion in Kidney Transplantation Recipients
Source: Transpl Int. 2023 Jun 23;36:11329. doi: 10.3389/ti.2023.11329 (PMC10326287; doi:10.3389/ti.2023.11329)
Supplement: Supplementary file 5 [file Table3.DOCX]

**Table S3. Antibody panel 2**

|  | Antigen | Tag | Clone | Com. | Cat# | Lot# | Dilution |
| --- | --- | --- | --- | --- | --- | --- | --- |
| 1 | CD45 | *89y* | HI30 | FLM | 3089003B | 3031908 | 1:100 |
| 2 | HLA-DR | *115ln* | L243 | BioL | 307651 | B293412 | 1:100 |
| 3 | DC SIGN | 141 Pr | 120507 | R&D | MAB161-100 | EAH0519031 | 1:100 |
| 4 | CD33 | 142 Nd | WM53 | BioLegend | 303419 | B234988 | 1:200 |
| 5 | CD11c | 143 Nd | B-ly6 | BD | 555391 | 7264831 | 1:200 |
| 6 | Slan | 144 Nd |  | Miltenyi |  |  | 1:100 |
| 7 | CD303 | 145 Nd | AC144 | Miltenyi | 130-108-063 | 5200706148 | 1:100 |
| 8 | CD163 | 146 Nd | GHI/61 | BioLegend | 333602 | B273529 | 1:100 |
| 9 | CD141 | 147 Sm | 1A4 | BD | 559780 | 9123822 | 1:200 |
| 10 | CLEC9A | 148 Nd | 8F9 | BioLegend | 353802 | B265751 | 1:100 |
| 11 | CD85k | 149 Sm | ZM4.1 | Biolegend |  | B287539 | 1:100 |
| 12 | CD155 | 150 Nd | SKII.4 | BioLegend | 337602 | B241901 | 1:100 |
| 13 | CD38 | 151 Eu | HIT2 | BioLegend | 303535 | B199604 | 1:400 |
| 14 | CD123 | 152 Sm | 6H6 | BioLegend | 306027 | B248462 | 1:400 |
| 15 | CD56 | 153 Eu | HCD56 | BioLEgend | 318345 | B254024 | 1:200 |
| 16 | CD16 | 154 Sm | 3G8 | BioLegend | 302051 | B232936 | 1:400 |
| 17 | CD36 | 155 Gd | 5271 | FLM | 3155012B | 342002 | 1:200 |
| 18 | CD86 | 156 Gd | IT2.2 | FLM | 3156008B | 3021908 | 1:100 |
| 20 | CD3 | 158 Gd | UCHT1 | BioLegend | 300443 | B289272 | 1:400 |
| 20 | CD19 | 158 Gd | HIB19 | BioLegend | 302247 | B282462 | 1:200 |
| 21 | CD39 | 159 Tb | A1 | BioLegend | 328221 | B242203 | 1:400 |
| 22 | CCR2 (CD192) | 160 Dy | K036C2 | BioLegend | 357202 | B240747 | 1:400 |
| 23 | Vista | 161 Dy | D1L2G | CST |  | 5 | 1:100 |
| 24 | EP2 | 162 Gd | EPR8060(B) | Abcam |  | GR3276094 | 1:100 |
| 25 | CD85d | 163 Dy | 42D1 | BioLegend | 338704 | B286313 | 1:100 |
| 26 | CD14 | 164 Dy | M5E2 | BioLegend | 301843 | B264979 | 1:100 |
| 27 | CD40 | 165 Ho | 5C3 | FLM | 3165005B | 132006 | 1:100 |
| 28 | CD1c | 166 Er | L161 | BioLegend | 331502 | B265380 | 1:400 |
| 29 | NKp46 | 167 Er | 9E 2 | BioLegend | 331902 | B289616 | 1:400 |
| 30 | Axl | 168 Er | 108724 | R&D | MAB154-100 | GCV0719031 | 1:100 |
| 31 | CD64 | 169 Tm | 10.1 | BioLegend | 305029 | B285916 | 1:400 |
| 32 | MerTK | 170 Er | 590H11G1E3 | BioLegend | 367602 | B286271 | 1:100 |
| 33 | CX3CR1 | 171 Yb | 2A9-1 | BioLegend | 341602 | B255429 | 1:100 |
| 34 | CD206 | 172 Yb | 19,2 | BD | 555953 | 9017769 | 1:400 |
| 35 | CD90 | 173 Yb | 5E10 | FLM | 3173011B | 2131910 | 1:50 |
| 36 | CD112 | 174 Yb | TX31 | BioLegend | 337402 | B246333 | 1:100 |
| 37 | PD-L1 | 175 Lu | 29E.2A3 | FLM | 3175017B | 3481808 | 1:100 |
| 38 | PD-L2 | 176 Yb | 24F.10C12 | BioLegend | 329613 | B280635 | 1:100 |
| 39 | CD172 (SIRPa) | 198 Pt | SE5A5 | BioLegend | 323802 | B216946 | 1:200 |
| 40 | CD11b | 209Bi | ICRF44 | FLM | 3209003B | 2007295-27 | 1:100 |
