# Supplementary material for: Peripheral Blood Immune Cell Composition After Autologous MSC Infusion in Kidney Transplantation Recipients
Source: Transpl Int. 2023 Jun 23;36:11329. doi: 10.3389/ti.2023.11329 (PMC10326287; doi:10.3389/ti.2023.11329)
Supplement: Supplementary file 6 [file Table4.DOCX]

**Table S4. Significant clusters**

| Cluster | Expression | Cell type |
| --- | --- | --- |
| NK cell |  |  |
| 330 | CD45RA^+^CD7^+^CD56^+^CD11c^+^CD127^+^GATA-3^+^EOMES^+^Tbet^+^ | CD11c^+^CD127^+^ NK cell |
|  |  |  |
| Undefined lineage |  |  |
| 211­­­­ | KLRG1^+^CD161^+^GATA3^+^CD7^+^CD127^+^CD25^+^ | KLRG1^+^CD161^+^GATA3^+^CD7^+^CD127^+^CD25^+^ |
|  |  |  |
| B cell |  |  |
| 5 | HLA-DR^+^CD19^+^CD22^+^CD39^+^ | Class switched memory B cell-like |
| 44 | CD45RA^+^HLA-DR^+^CD19^+^CD21^+^CD22^+^CD27^+^CD39^+^ | Class switched CD11c-  memory B cell-like |
| 43 | CD45RA^+^HLA-DR^+^CD19^+^CD22^+^CD11c^+^CD27^+^CD39^+^ | Class switched CD11c^+^  memory B cell-like |
| 47 | CD45RA^+^HLA-DR^+^CD19^+^CD21^+^CD22^+^CD11c^+^CD39^+^  Ki-67^+^ | Proliferating CD11c^+^ B cell-like |
| 13 | CD19^+^CD21^+^CD22^+^ | Memory B cell-like |
| 28 | HLA-DR^+^CD19^+^CD21^+^CD22^+^IgM^+^CD38^+^CCR7^+^ | CCR7^+^CD38^+^ mature B cells-like |
|  |  |  |
| CD8 |  |  |
| 196 | CD45RA^+^CD45RO^+^CD3^+^CD8^+^CD7^+^CD57^+^KLRG1^+^EOMES^+^Tbet^+^ | CD57^+^CD45RA^+^CD45RO^+^ Tc1-like cytotoxic T cell |
| 263 | CD45RO^+^CD3^+^CD8^+^CD7^+^CD27^+^CD127^+^EOMES^+^Tbet^+^ | CD27^+^CD127^+^ memory Tc1-like cytotoxic T cell |
| 258 | CD45RO^+^CD3^+^CD8^+^CD7^+^CD27^+^CD39^+^CD127^+^Tbet^+^ | CD39^+^CD27^+^CD127^+^ memory Tc1-like cytotoxic T cell |
| 255 | CD45RO^+^CD3^+^CD8^+^CD5^+^CD7^+^CD27^+^CD57^+^CD127^+^KLRG1^+^EOMES^+^Tbet^+^ | CD27^+^CD57^+^CD127^+^ memory Tc1-like cytotoxic T cell |
| 252 | CD45RO^+^CD3^+^CD8^+^CD5^+^CD7^+^CD27^+^CD57^+^CD127^+^KLRG1^+^EOMES^+^Tbet^+^Ki-67^+^ | Proliferating CD27^+^CD57^+^CD127^+^  memory Tc1-like cytotoxic T cell |
| 253 | CD45RO^+^CD3^+^CD8^+^CD5^+^CD7^+^CD27^+^CD57^+^KLRG1^+^PD-1^+^Tigit^+^EOMES^+^Tbet^+^Ki-67^+^ | Proliferating CD27^+^CD57^+^CD127^-^PD-1^+^Tigit^+^ memory Tc1-like cytotoxic T cell |
| 254 | CD45RO^+^CD3^+^CD8^+^CD5^+^CD7^+^CD57^+^CD127^+^KLRG1^+^EOMES^+^Tbet^+^ | CD57^+^CD127^+^ effector  memory Tc1-like cytotoxic T cell |
| 259 | CD45RO^+^CD3^+^CD8^+^CD5^+^CD7^+^CD127^+^GATA-3^+^Tbet^+^ | CD57^-^CD127^+^ effector memory  Tc1/Tc2-like cytotoxic T cell |
| 96 | CD45RO^+^HLA-DR^+^CD3^+^CD4^+^CD5^+^CD127^+^GATA-3^+^Ki-67^+^ | Proliferating HLA-DR^+^CD7-CD127^+^ effector memory Th2-like |
|  |  |  |
| CD4 Th2 |  |  |
| 141 | CD45RO^+^CD3^+^CD4^+^CD5^+^CD7^+^CD27^+^CD127^+^CCR7^+^GATA-3^+^ | CD7^+^CD27^+^CD127^+^ central memory Th2-like |
| 149 | CD45RO^+^CD3^+^CD4^+^CD5^+^CD7^+^CD27^+^CD127^+^GATA-3^+^ | CD7^+^CD27^+^CD127^+^ effector memory Th2-like |
| 151 | CD45RO^+^CD3^+^CD4^+^CD5^+^CD7^lo^CD27^+^CD127^+^GATA-3^+^ | CD7^lo^CD27^+^CD127^+^ effector memory Th2-like |
| 228 | CD45RO^+^CD3^+^CD4^+^CD5^+^CD127^+^GATA-3^+^ | CD7^-^CD127^+^ effector memory Th2-like |
| 224 | CD45RO^+^CD3^+^CD4^+^CD127^+^CD161^+^ | CD127^+^CD161^+^ effector memory Th2-like |
| 220 | CD3^+^CD4^+^CD5^+^CD39^+^CD127^+^GATA-3^+^ | CD39^+^CD7^-^CD127^+^ effector memory Th2-like |
| 152 | CD45RO^+^CD3^+^CD4^+^CD5^+^CD27^+^PD-1^+^GATA-3^+^ | CD27^+^CD127^-^PD-1^+^ effector memory Th2-like |
| 96 | CD45RO^+^HLA-DR^+^CD3^+^CD4^+^CD5^+^ CD127^+^GATA-3^+^Ki-67^+^ | Proliferating HLA-DR^+^CD7^-^CD127^+^ effector memory Th2-like |
|  |  |  |
| Activated CD4 Th2 |  |  |
| ­­229 | CD45RO^+^CD3^+^CD4^+^CD5^+^CD127^+^GATA-3^+^ | CD7^+^CD127^+^ activated effector memory Th2-like |
| 145 | CD45RO^+^CD3^+^CD4^+^CD5^+^CD7^+^CD27^+^CD127^+^CD25^+^GATA-3^+^ | CD7^+^CD27^+^CD127^+^ activated effector memory Th2-like |
| 148 | CD45RO^+^CD3^+^CD4^+^CD5^+^CD7^+^CD27^+^CD57^+^CD127^+^CD25^+^GATA-3^+^ | CD7^-^CD27^+^CD127^+^ activated  effector memory Th2-like |
| 226 | CD45RO^+^CD3^+^CD4^+^CD5^+^CD127^+^CD25^+^GATA-3^+^ | CD7^-^CD127^+^ activated  effector memory Th2-like |
| 223 | CD45RO^+^CD3^+^CD4^+^CD5^+^CD7^+^CD127^+^CD161^+^PD-1^+^CD25^+^GATA-3^+^ | CD127^+^CD161^+^PD-1^+^ activated  effector memory Th2-like |
| 227 | CD45RO^+^CD3^+^CD4^+^CD5^+^CD7^+^CD27^+^CD25^+^GATA-3^+^ | CD7^+^CD27^+^ activated effector memory Th2-like |
|  |  |  |
| CD4 Th1/Th2 |  |  |
| 217 | CD45RO^+^CD3^+^CD4^+^CD5^+^CD57^+^CD127^+^PD-1^+^GATA-3^+^Tbet^+^ | CD57^+^CD127^+^PD-1^+^ effector memory Th1/Th2-like |
| 222 | CD45RO^+^CD3^+^CD4^+^CD5^+^CD57^+^CD127^+^  GATA-3^+^Tbet^+^Ki-67^+^ | Proliferating CD57^+^CD127^+^ effector memory Th1/Th2-like |
| 221 | CD45RO^+^HLA-DR^+^CD3^+^CD4^+^CD5^+^CD7^+^CD39^+^CD57^+^CD127^+^GATA-3^+^Tbet^+^Ki-67 | Proliferating HLA-DR^+^CD39^+^CD57^+^CD127^+^ effector memory Th1/Th2-like |
|  |  |  |
| Treg |  |  |
| 133 | CD45RO^+^CD3^+^CD4^+^CD5^+^CD27^+^CD39^+^CD25^+^GATA-3^+^FoxP3^+^ | FoxP3^+^CD7^-^TIGIT^-^CTLA-4^-^CD39^+^ Treg |
